# Unravelling adolescent girls’ aspirations in Nepal: Status and associations with individual-, household-, and community-level characteristics

**DOI:** 10.1371/journal.pone.0258416

**Published:** 2021-11-12

**Authors:** Dónya S. Madjdian, Kenda Cunningham, Hilde Bras, Maria Koelen, Lenneke Vaandrager, Ramesh P. Adhikari, Elise F. Talsma

**Affiliations:** 1 Health & Society, Department of Social Sciences, Wageningen University & Research, Wageningen, The Netherlands; 2 Division of Human Nutrition and Health, Wageningen University & Research, Wageningen, The Netherlands; 3 Helen Keller International, New York, NY, United States of America; 4 Faculty of Epidemiology and Population Health, Department of Population Health, London School of Hygiene and Tropical Medicine, London, United Kingdom; 5 Economic and Social History, Department of History, University of Groningen, Groningen, The Netherlands; 6 Helen Keller International, Kathmandu, Nepal; University of Botswana, BOTSWANA

## Abstract

**Background:**

Adolescents’ aspirations have potential to influence their present and future well-being. Limited knowledge exists on adolescent girls’ aspirations and their determinants, particularly in low-income contexts.

**Methods and findings:**

Using cross-sectional data, collected in 2018 in Nepal, within the *Suaahara II* Adolescent Panel Survey, (n = 840), adolescent girls’ aspirations in several domains—education, occupation, marriage, fertility, health, and nutrition–were described. Regression models were estimated to explore associations between individual, household and community characteristics and these aspirations for all adolescents and separately for younger (10–14 years) and older (15–19 years) girls. Age, school attendance, and self-efficacy, as well as household wealth, caste/ethnicity, size, and agro-ecological zone of residence were significantly associated with aspirations, although effect sizes and significance varied by aspiration domain and age group.

**Conclusions:**

Findings underscore the curtailing effect of poverty on aspirations and the dynamic nature of aspirations. Initiatives to foster girls’ aspirations must address both individual and contextual factors.

## Introduction

Adolescence (ages 10–19 years), a time of rapid change in several life domains, has gained increased interest, particularly in low- and middle- income countries (LMICs) where roughly 90% of the world’s adolescents live [1, 2]. As part of non-cognitive and socio-emotional skill development, adolescence is a key time for the development of aspirations [3]. Aspirations influence behavioural choices and exposure to factors and thus, may be important drivers of life outcomes, including health and wellbeing, nutrition, reproductive health, labour participation, and education [4–9].

The development of girls’ aspirations related to education, occupation, family formation (i.e. marriage, fertility), health, and nutrition is dynamic (i.e., non-linear) and influenced by girls’ capacity to aspire, their life stage and age, as well as a range of internal and external factors [10, 11]. In many South-Asian contexts, transitions through adolescence are influenced by deeply-rooted gender norms that regulate how girls should act or what they should become [10, 11], and consequently, what they (can) aspire for. For instance, in India, data from a cross-sectional survey among adolescent girls aged 10–19 years, showed that married and out-of-school girls were expected to prepare, and consequently aspire for, a role as homemaker and being a mother, rather than aspiring for an occupation [11].

So far, research on the nature, scope and drivers of adolescent girls’ aspirations lacks in four areas. First, most studies are characterized by small sample size or focus on specific sub-groups, such as married or older adolescents [6], rather than all adolescents, preventing comparisons between the different stages of adolescence. Second, only a few studies on adolescent aspirations have been conducted in low-income contexts and studies from South-Asia in particular, are scarce [6]. Third, the majority of studies focus on educational and occupational aspirations, but not on aspirations in other important life domains including family formation and one’s health and nutritional wellbeing [6, 12–15]. Fourth, studies are commonly devoted to single factors associated with aspirations, such as age, ethnicity, socio-economic status, or non-cognitive personal traits, but studies examining the associations between various factors at the individual, household, and community levels and the different domains of adolescent girls’ aspirations are scant [6, 16].

Therefore, this paper’s contribution to the body of knowledge on adolescent girls’ aspirations and their drivers in the South-Asian context of Nepal is threefold. First, the study aims to assess six domains of aspirations, rather than focusing on a selection of aspirations. Secondly, it aims to explore a range of individual, household, and community factors that may be associated with each of these aspirations. And third, the study advances on previous research by exploring variations in the associations between younger (10–14 years) and older (15–19 years) girls. In Nepal, undernutrition, early marriage and childbearing, incomplete schooling, unequal work opportunities, and limited decision-making power within and outside households remain significant challenges for Nepalese adolescent girls [17]. These challenges play out as inequalities between boys and girls and may affect aspirations for future lives. For instance, an older study in Nepal showed a gender gap in aspirations with boys having wider aspirations related to education, work, marriage and childbearing than girls, as well as having more opportunities to realize these aspirations [4]. Today, despite achieving almost gender parity in primary education, Nepali girls lag behind boys in terms of learning outcomes and access to and enrolment in quality, particularly secondary education [18, 19]. Girls are also more likely than boys to repeat classes across all grades and are less likely to pass their school leaving certificate. Moreover, girls have less labour market opportunities, which, combined with household poverty or social pressure, might force girls to marry rather than continue their studies [20]. Unequal access to health care, and skewed intrahousehold food allocation practices leading to food and micronutrient deprivation, according to age, or sex are persistent [21]. At the time of the study, data also showed that the percentage of girls marrying before their 18^th^ birthday is declining. However, still 27.1% of girls marries between the ages of 15 and 19 years, compared to 6.4% of boys. And, almost a third of married girls has given birth to a first child, by the age of 19 [22]. A recent review on girls’ psychosocial wellbeing in Nepal showed that many girls felt depressed, sad, lacked confidence, or felt they could not cope with their current living situation [23]. Yet, limited evidence exists on Nepalese adolescent girls’ aspirations in education, occupation, marriage, fertility, health and nutrition; drivers of these aspirations; and how these differ during different stages of adolescence. Such knowledge is essential to inform programs and policies to support girls in reaching their full potential.

Using data from the *Suaahara* II adolescent panel survey, we measure Nepalese adolescent girls’ aspirations in several key life course domains—education, occupation, marriage, fertility, health, and nutrition -, estimate associations between differing aspiration domains and a range of individual, household, and community factors, and estimate variations in these associations between younger (ages 10–14) and older (ages 15–19) adolescent girls. While we acknowledge that aspirations in life domains may be interdependent, for the purpose of this study we look at the determinants of each individual aspiration rather than assessing the interrelations between aspirations.

### Conceptualising aspirations

In this study, we use Hart’s [5] definition of aspirations as “future-oriented, driven by conscious and unconscious motivations” and “indicative of an individual’s or group’s commitments towards a particular trajectory or end point” [5]. This definition assumes that individuals desire to do well and that aspirations do not develop in isolation [5, 24]. It moreover acknowledges the role of human agency, the dynamic nature of aspirations, and the influence of internal and external factors.

This study also draws upon Appadurai’s capacity to aspire theory that notes how one’s capacity to aspire is not equally distributed across society but rather shaped by context [25]. Thus, poverty is theorized to be one of the biggest external constraints; people born in poverty aspire less [25]. Agency, one’s freedom to “pursue goals with influence beyond oneself and that one has reason to value” is inherently linked to the capacity to aspire [5, 12, 24, 26]. Particularly during adolescence, girls’ sense of agency, and their decision-making power in important life domains fluctuates, which may influence the freedom to use resources and their aspirations. The extent to which they are able to do so, also depends on context-specific opportunities and restrictions in girls’ everyday lives [27–29]. Aspirations are dynamic and likely to change throughout adolescence, following certain life events, and transitions [6]. For instance, early marriage and subsequent school drop-out change one’s social status and often affect a girl’s later participation in the workforce [30]. Other structural, socio-cultural and economic factors may either hinder or enable aspirations [10]. The remainder of this section expands on the individual, household, and community factors that likely influence adolescent girls’ aspirations in the context of Nepal.

#### Individual factors and aspirations

Individual factors that may relate to girls’ aspirations include her age and life events, such as leaving school or becoming a mother, self-efficacy and decision-making power [6, 12, 31]. Compared to boys, girls in LMICs often see their worlds and dreams narrowing as from early adolescence onwards [10]. Two reviews showed that with increasing age, and often upon social status transitions, girls tend to become more pessimistic and adjust their aspirations downwards to reflect reality or other’s expectations [6, 13]. For instance, a qualitative study in the plains of Nepal found that adolescent girls aged 10–15 years, generally felt submissive and were unable to express their preferences, hindering growth and articulation of aspirations [32]. A qualitative study in Mozambique with girls aged 13–19 years, also showed that younger girls (13–16 years) generally seemed more hopeful and felt more on track towards reaching their goals [33]. Hence, with age, and with a naturally occurring shift from being present-focused to becoming more future-oriented as part of adolescent development, aspirations may be adjusted towards social expectations, perceived constraints, especially in poor-resource contexts.

Additionally, important life events often imply social transitions. For instance, menarche signifies a transition from being a girl to becoming a woman, often marking marital readiness. Although the legal age of marriage is 20 years, about 37% of Nepalese girls marry before age 18 and first pregnancy follows often within one or two years [22]. In Nepal’s predominantly patriarchal society, the need to conform to socially-expected roles and norms connected to a girl’s new status leads them to reconsider their aspirations [12, 23, 34, 35]. Contrarily, school enrolment may delay the pressure to marry, in turn influencing marital and childbearing aspirations [36]. Marriage and motherhood might change one’s occupational aspirations, for instance when husbands or in-laws limit girls’ decision-making power [37]. To our knowledge, no studies have focused specifically on aspirations surrounding health or nutrition among adolescents in LMICs. Unequal intra-household allocation of food and health care favouring men, however, is still a pressing problem for older adolescent girls and young women in Nepal [38, 39], potentially affecting perceived importance of health and nutrition, and consequently girls’ aspirations in these domains.

Self-efficacy is a psychological dimension of agency and seen as an important regulator of aspirations [40]. Indicating the extent to which a girl feels in charge of her (future) life, self-efficacy has been positively associated with occupational [41] and educational [42, 43] aspirations, but less so with other aspirations such as those related to her diet or health. Limited agency in these areas might reduce perceived importance of, and access to health and nutrition [39, 44]. Decreased self-efficacy results in a sense of fatalism or hopelessness lowering aspirations and in turn, negatively impacts human development [45]. This is of concern in the context of Nepal, where many girls still report low self-efficacy [23, 46]. Similarly, girls’ decision-making power related to their lives either fosters or restricts their aspirations [47].

#### Household factors and aspirations

Poverty has been extensively studied in relation to aspirations. Evidence is, however, conflicting. The ‘poverty of aspirations’ hypothesis suggests that poverty is the main reason for having no or low aspirations. Living in poverty, it is suggested, reduces hope, which results in a so-called ‘aspirations failure’. This failure, or a fatalistic attitude, may reinforce poverty [24, 25]. Others, however, argue that poorer children’s aspirations are as high as, or even higher than children from wealthier households [6, 48, 49]. A qualitative study on aspirations of adolescent girls (ages 15–19) in Jumla, a remote mountain district in Far-Western Nepal, highlighted poverty as the main barrier to educational aspirations, even more so for marginalized or socially-excluded groups [36].

Household composition may affect girls’ aspirations. Living within larger families may generate conflicts, as limited household resources are divided amongst more members. A cross-sectional study in India showed that girls (15–24 years) with fewer siblings preferred a small family size [50]. Prior studies have also found associations between other proxies of socio-economic status, such as parental occupation and educational levels, and adolescents’ aspirations [51, 52]. Moreover, in Nepal the increase in female-headed households due to male emigration for work is accompanied by shifts in identity, agency, and labour division [53]. While these households tend to be economically worse off than male-headed households, they have also been associated with higher female bargaining power within households, leading to better health, nutritional, and educational outcomes of children, sometimes despite poverty [54–57]. As a consequence Nepalese girls’ aspirations may be changing, as the context is changing. Other factors such as caste/ethnicity may affect aspirations via internalised values that perpetuate inequalities [5]. In Nepal, where caste/ethnicity have long been linked to access and socio-economic disparities, caste/ethnicity might be an important determinant of aspirations [3].

#### Community factors and aspirations

Aspirations are located within existing structures and develop in cultural and institutional contexts [58]. The geographic location in which an adolescent girl grows up might affect her aspirations through context-specific, structural opportunities related to girls’ life trajectories in domains of education, occupation, family formation, and health and nutrition. In other words, the community (place, habitation) in which adolescents grow up may affect their aspirations [6]. In addition to Nepal’s hundreds of caste/ethnic and linguistic groups, communities span from lowland plains to some of the tallest mountains in the world. Opportunities for education, labour participation, family formation, and access to health care and nutritious foods differ by Nepal’s geography. For instance, undernutrition and food insecurity prevalence, as well as the percentage of girls that marry early and the percentage of girls dropping out of school is higher in the plains (*Terai*) than in other regions of Nepal [59]. Previous research also reported lower health care use and health outcomes in this region, possibly due to limited health and education services [60, 61]. Living in mountainous or hilly areas may also challenge adolescents’ livelihoods due to the remoteness and lack of infrastructure. Moreover, the country is facing rapid urbanization and increased migration-flows [62]. These and other changes create variation in access to information and health care, educational services, and markets [63]. Within-country differences in girls’ aspirations are therefore highly likely.

## Methods

### Data and sample

Data for this study were drawn from an Adolescent Girls Panel collected under the USAID-funded *Suaahara II* project and its adolescent learning agenda, representative of 42 of Nepal’s 77 districts where this integrated nutrition program (2017–2023) is implemented. The cohort study aims to chart the lives of 1093 adolescent girls, who were 10–19 years in 2017, as they transition through adolescence and into adulthood.

Multi-stage cluster sampling, with probability proportional to size sampling techniques, was used to select districts (n = 16), one rural and urban municipality per district (n = 32), three wards per municipality (n = 96), and two clusters per ward (n = 192). For the last stage, a household census was conducted in each cluster, and from the census, 19 households with a child 0–5 years and their (residing) mother were randomly selected based on the number of required households (n = 3648) resulting from sample size and power calculations to measure changes in indicates of interest for the Suaahara II program. If an adolescent girl (10–19 years) resided in the selected household, she was also invited to participate in the survey (n = 837). If more than one adolescent girl resided in the household, one was randomly included. Some of the participants (n = 256) randomly selected for the interview as part of the mother’s questionnaire were also of adolescent age (15–19 years). These adolescents were all combined as a cohort to be followed annually resulting in a total sample of 1093 adolescent girls in 2017. For more information on the sampling approach, we refer to Cunningham and colleagues [46]. For this paper, we use data from the first follow-up survey (July 2—September 14, 2018), as this wave was the first to include a module on adolescent aspirations. Trained female enumerators collected data in each adolescent’s home, using survey questionnaires that had been pilot-tested, translated and back translated, and programmed on mobile phones using Open Data Kit. In this wave, 975 out of 1093 adolescent girls aged 10 to 21 participated. The loss to follow-up rate of 11% can be mostly attributed to migration, usually for education, marriage, or work. We excluded those who were 20–21 years and no longer adolescents (n = 131) and who did not answer the aspirations questions (n = 4). Our total analytical sample is 397 younger adolescent girls (10–14 years) and 443 older adolescent girls (15–19 years) (Total N = 840).

### Measures

#### Dependent variables

Study outcomes cover aspirations on six life course domains: education, occupation, marriage, fertility, health, and nutrition. Questions were adapted from existing surveys or methods that have been used previously for aspiration or goal assessment, including the Young Lives study component on educational and occupational aspirations [64], the Aspiration Index for health and nutrition aspirations [65], and the Demographic and Health Surveys (DHS) for family formation aspirations [66]. Girls’ educational aspirations were indicated by their aspired grade/level (class 1 to MSc level, stopping schooling or ‘don’t know’). This variable was dichotomized into aspiring to study up to and including class 12 (including stopping now, or don’t know, = 0), or aspiring to study beyond class 12 (= 1). Occupational aspirations were indicated by girls’ aspired job. Job types were categorized into according to skill-levels (0–4) based on the Nepal Standard Classification of Occupations (NSCO) [59], and then dichotomized into aspiring for no job or a job requiring less skills (= 0), versus aspiring for a job requiring professional skills or higher education (= 1). Marital and fertility aspirations were indicated by aspired age of first marriage, and aspired age of having a first child, respectively. Health and nutrition- related aspirations were measured by perceived importance of health and nutrition, scored on a 5-point scale (1 = not important at all, 5 = very important).

All questions focused on aspirations in the absence of any constraints (i.e. “imagine you could study for as long as you wanted…”) and at that specific moment in time. Questions were tailored to the girls’ specific life situations through skip patterns. For example, out-of-school girls were not asked about their educational aspirations, married girls were not asked about their aspired age of marriage, and mothers were not asked about their aspired age of having a first child. Among the six domains, “don’t know” was allowed as a response for educational, occupational, and health- and nutrition- related aspirations. We excluded girls with uncertain or undecided marital (n = 48) or fertility aspirations (n = 299) and those who wished for no children (n = 10) in specific models related to those outcomes. Table 1 provides a detailed overview of the dependent variables, their respective categorizations, and sample sizes.

**Table 1:** Dependent variables: Definitions, survey questions, and sample sizes.

Aspirations	Question	Answer options	Classification	Sample
Education^a^	Imagine you could study for as long as you liked, until what grade/level would you wish to be in school?	1–10: Class 1–10 (SLC/SEE)^a^	0 = ≤class 12 (Ref)	Only school-going girls (n = 630)
		11: Class 11	(1–12, 15, 98)	
		12: Class 12 (+2)	1 = >class 12	
		13: Bachelors	(13–15)	
		14: MSc or higher		
		15: Stop now		
		98: Don’t know		
Occupation^b^	Imagine you could do whatever you would like to do, what (type of) job would you like to have in your future?	1: Agriculture/fishery	0 = no job, skill-level 1 and 2 (Ref)	All girls (n = 840)
		2: Education		
		3: Health professional		
		4: Housewife	(1, 4, 9–10, 12, 14–16, 98)	
		5: Business, sales		
		6: IT	1 = skill-level 3&4	
		8: Government job		
		9: Service work	(2, 3, 5, 6, 8, 11)	
		10: Industry/plant work		
		11: Engineer		
		12: No job/unemployed		
		14: Artist/handicraft		
		15: Beautician		
		16: Tailor		
		96: Other (specified & categorized)		
		98: Don’t know		
Marriage	Imagine you could marry whenever you wished, at what age would you like to get married?	Age in years	Scale (x to y)	Only unmarried girls excluding those with uncertain/undecided aspirations (n = 613)
		98: Don’t know		
Fertility	Imagine you could have your first child whenever you wished, at what age would you wish to have your first child?	Age in years	Scale (x to y)	Only girls without a child(ren) excluding those with uncertain/undecided aspirations or those who did not wish to have children (n = 381)
		98: Don’t know		
Health	How important is it for you to be physically healthy?	1: Not important at all	0 = Else (1–4, 98) (Ref)	All girls (n = 840)
		2: Not really important		
		3: Neutral	1 = Very important (5)	
		4: Somewhat important		
		5: Very important		
		98: Don’t know		
Nutrition	How important is it for you to eat nutritious food?	1: Not important at all	0 = Else (1–4, 98) (Ref)	All girls (n = 840)
		2: Not really important		
		3: Neutral	1 = Very important (5)	
		4: Somewhat important		
		5: Very important		
		98: Don’t know		

^a^According to Nepal’s new Education Act, the Secondary Education Examination (SEE), previously SLC (School Leaving Certificate), is taken in the 10^th^ grade, and before joining higher secondary or intermediate level education (12th grade).

^b^According to the Nepal Standard Classification of Occupations (NSCO) [67]

#### Independent variables

Our independent variables include individual-, household-, and community-level characteristics. Individual characteristics included the girls’ age group, school-going status, maternal status, self-efficacy, and decision-making power. Binary variables were created for age (10–14 years vs. 15–19 years), school status (in- vs. out-of-school), and maternal status (mother vs. not a mother). Self-efficacy scores were measured using the 8-item New General Self-Efficacy Scale [60]. Using a 5-point scoring scale (1 = strongly disagree and 5 = strongly agree), girls indicated how much they agreed with each statement. The scale was found to be reliable for both age groups (α = 0,81 and α = 0,82 respectively). Decision-making was measured based on four questions relating to a girl’s perceived input in decisions regarding continuing school, going out of the house to engage in the community, food consumption, and health care, using a 3-point rating scale (1 = little to no input and 3 = input in most or all decisions). Binary variables were then created (none or some input vs. input into most or all decisions) for each of the four questions, due to low frequencies in the none or some input categories.

Household characteristics included size, economic status, caste/ethnicity, and food security, as well as the household head’s sex, and level of education. Household size was measured as a continuous variable and captured the total number of people that were usually resident in the household. Household economic status was measured by calculating total equity scores, using the NDHS 2016 equity tool which sums the number of consumer goods owned (television(s), cupboard(s), table(s) and fan(s)), source of energy for cooking, and quality of housing materials (roof, floor, and wall) [68]. Caste/ethnicity included three categories: socially excluded (i.e. *Dalit*, *Muslim*, disadvantaged *Janajati*); upper caste (i.e. *Brahmin* and *Chettri)*, and others (i.e. *Newar*, *Gurung*, *Thakali*, non-*Dalit terai* caste). Household food security was measured using the Household Food Insecurity Access Scale; a binary variable was created (food secure vs. food insecure) [69]. The sex of the household head was created as a binary variable (male vs. female) and his/her level of (formal) education categorized into none, some primary (grade 1–5), or at least some secondary (class 6 or beyond).

Community characteristics included a categorical variable for agro-ecological zone of residence (mountains, hills, and plains) and a binary variable to denote whether the community was disadvantaged or not, based on a previous classification by the Government of Nepal as category 3A, 3B and 4 (versus 1 or 2), due to its remoteness, food insecurity, and other factors [70].

### Analyses

Analyses were conducted using STATA v.15 (StataCorp). Descriptive analyses of sample characteristics and bivariate analyses, including unadjusted Chi-square and Pearson’s correlation tests to detect significant differences in all aspirations by the independent variables were done (Table 2). Logistic and linear regressions were conducted, using dummy variables for all categorical independent variables. Binomial logistic regressions were estimated for educational and occupational aspirations and linear regression for fertility and marital aspirations (Table 3). No regression analyses were run for health and nutrition aspirations, as about 90% of girls reported very high health and nutrition aspirations and this would have resulted in quasi-complete separation issues. We also included regression analyses by age group (younger vs. older girls) for educational, occupation and marital aspirations (Table 4). We did not estimate differences between the two age groups for fertility aspirations as the majority (69%) of younger girls reported to be uncertain/undecided about their fertility aspirations. Variance inflation factors (VIF) were calculated and no multicollinearity was found between independent variables. Regression models were adjusted for clustering at the primary sampling unit. Regression results are reported as adjusted odds ratios (aOR), controlling for other independent variables in the model for binary logistic regressions, and unstandardized regression coefficients (*B*), as well as standardized beta coefficients for continuous variables, with 95% confidence intervals for linear regressions. Statistical significance was considered at p<0.05, p<0.01 and p<0.001 levels.

**Table 2:** Descriptive and correlation statistics.

			Educational aspirations		Occupational aspirations		Marital aspirations		Fertility aspirations		Health aspirations		Nutrition aspirations	
*Total observations*		*840*	*630*		*840*		*613*		*381*		*840*		*840*	
			*Beyond class 12*		*Professional-skilled job*		*Mean age*		*Mean age*		*Important*		*Important*	
			*(vs*. *lower than class 12) *		*(vs*. *no*, *or less-skilled job)*						*(vs*. *not important)*		*(vs*. *not important)*	
**N % or/ mean (SD)**	* *		369 (58.6)	*p*	617 (73.5)	*p*	22.3 (2.3)	*p*	24.3 (2.7)	* p*	758 (90.2)	*p *	872 (90.1)	*p*
**Age group**	*Young*	397 (47.3)	201 (52.2)	<0.000	331 (83.4)	<0.000	22.0 (2.3)	0.003	24.5 (2.7)	0.178	356 (89.7)	0.601	348 (87.7)	0.057
	*Older*	443 (52.7)	168 (68.6)		286 (64.6)		22.6 (2.3)		24.1 (2.7)		402 (90.7)		406 (91.6)	
**Maternal status**	*Not a mother*	690 (82.1)	365 (58.6)		558 (80.8)	<0.000	n/a		n/a		624 (90.4)	0.680	616 (89.3)	0.318
	*Mother*	150 (17.9)	n/a		59 (39.3)		n/a		n/a		134 (89.3)		138 (92.0)	
**School status**	*In-school*	630 (75.0)	369 (58.6)		536 (85.1)	<0.000	22.3 (2.3)	0.162	24.6 (2.6)	<0.000	569 (90.3)	0.893	566 (89.8)	0.895
	*Out of school*	210 (25.0)	n/a		81 (38.6)		21.8 (1.9)		22.1 (2.6)		189 (90.0)		188 (89.5)	
**School decision-making input**	*None to some input*	307 (36.5)	52 (45.2)	0.001	n/a		n/a		n/a		n/a		n/a	
	*A lot of input*	533 (63.5)	317 (61.6)		n/a		n/a		n/a		n/a		n/a	
**Going out decision-making input**	*None to some input*	750 (89.3)	n/a		561 (74.8)	0.011	n/a		n/a		n/a		n/a	
	*A lot of input*	90 (10.7)	n/a		56 (62.2)		n/a		n/a		n/a		n/a	
**Health decision-making input**	*None to some input*	257 (30.6)	n/a		n/a		n/a		n/a		222 (86.4)	0.012	n/a	
	*A lot of input*	583 (69.4)	n/a		n/a		n/a		n/a		536 (91.9)		n/a	
**Food decision-making input**	*None to some input*	489 (58.2)	n/a		n/a		n/a		n/a		n/a		424 (86.7)	0.001
	*A lot of input*	351 (41.8)	n/a		n/a		n/a		n/a		n/a		330 (94.0)	
**Self-efficacy**	*Score*	30.5 (4.6)	31.8 (4.3)	<0.000	31.0 (4.5)	<0.000	22.3 (2.3)	0.008	24.3 (2.7)	0.109	30.6 (4.6)	0.093	30.7 (4.6)	0.008
**Caste/ethnicity**	*Socially-excluded*	442 (52.6)	173 (54.2)	0.007	307 (69.5)	0.021	22.2 (2.4)	0.689	24.3 (2.7)	0.324	397 (89.8)	0.753	387 (87.5)	0.069
	*Brahmin/ Chettri*	327 (38.9)	168 (65.9)		254 (77.7)		22.4 (2.3)		24.5 (2.7)		298 (91.1)		303 (92.7)	
	*Other*	71 (8.5)	28 (50.0)		56 (78.9)		21.9 (2.1)		23.4 (2.6)		63 (88.7)		64 (90.1)	
**Household size**	*Number*	6.2 (3.0)	6.1 (2.7)	0.212	6.2 (2.7)	0.094	22.3 (2.3)	0.089	24.3 (2.6)	0.071	6.2 (3.0)	0.767	6.1 (3.0)	0.448
**Household wealth**	*Score*	-0.15 (1.16)	0.02 (1.24)	<0.000	-0.1 (1.12)	0.043	22.3 (2.3)	<0.000	24.3 (2.6)	0.010	-0.1 (1.2)	0.001	-0.1 (1.2)	0.029
**Head of household sex**	*Female*	392 (46.7)	125 (42.8)	0.513	246 (84.2)	0.586	22.3 (2.3)	0,741	24.6 (2.8)	0.025	262 (89.7)	0.641	257 (88.0)	0.158
	*Male*	448 (53.3)	202 (59.8)		290 (85.8)		22.2 (2.3)		24.0 (2.6)		307 (90.8)		309 (91.4)	
**Head of household highest education completed**	*No (formal) education*	355 (42.3)	133 (52.8)	0.040	205 (81.4)	0.098	22.2 (2.4)	0.207	24.0 (2.8)	0.100	225 (89.3)	0.755	222 (88.1)	0.423
	*Primary*	221 (26.3)	105 (60.3)		153 (87.9)		22.3 (2.3)		24.3 (2.5)		159 (91.4)		160 (91.9)	
	*Secondary or higher*	264 (31.4)	131(64.2)		178 (87.2)		22.4 (4)		24.6 (2.7)		185 (90.7)		184 (90.2)	
**Household food security**	*Secure*	322 (38.3)	240 (62.0)	0.027	336 (86.8)	0.121	22.5 (2.3)	0.004	24.4 (2.7)	0.400	358 (92.5)	0.019	353 (91.2)	0.150
	*Insecure*	518 (61.7)	129 (53.1)		200 (82.3)		21.9 (2.4)		24.1 (2.7)		211 (86.8)		213 (87.7)	
**Agro-ecological zone**	*Mountains*	122 (14.5)	65 (61.9)	0.494	98 (80.3)	0.166	22.0 (2.4)	0.034	24.2 (2.8)	0.940	109 (89.3)	0.064	112 (91.8)	0.645
	*Hills*	426 (50.7)	184 (59.6)		310 (72.8)		22.2 (2.3)		24.4 (2.6)		376 (88.3)		379 (89.0)	
	*Terai*	292 (34.8)	120 (55.6)		209 (71.6)		22.5 (2.3)		24.2 (2.8)		273 (93.5)		263 (9012)	
**Disadvantaged community**	*Yes*	272 (32.4)	103 (51.0)	0.008	164 (81.2)	0.060	22.5 (2.3)	0.001	23.8 (2.6)	0.020	242 (88.9)	0.392	234 (86.0)	0.014
	*No*	568 (67.6)	266 (62.1)	372 (86.9)		21.8 (2.3)		24.5 (2.7)		516 (90.8)		520 (91.5)	

*n/a indicates not included in analysis

**Table 3:** Regression results for educational, occupational, marital, fertility, health, and nutrition aspirations.

Aspirations			Education		Occupation		Marriage			Fertility	
		N	> class 12 (1)	N	Professional skilled job (1)	N	Age		N	Age	
			vs.								
					vs. No/low skilled job (0)						
			≤class 12 (0)								
Total observations		630		840		613			381		
			aOR (95% CI)		aOR (95%CI)		Unstd. B (95% CI)	Std. Beta (95%CI)		B (95% CI)	Beta
**Age Group**	*15–19 years*	245	1.73 (1.19–2.53)**	443	0.89 (0.55–1.42)	255	0.52 (0.16–0.88)**	0.52 (0.16–0.88)**	195	-0.04 (-0.51–0.43)	-0.04 (-0.51–0.43)
(ref = 10–14 years old)											
**Maternal status**	*Mother*	n/a	n/a	150	0.92 (0.46–1.79)	n/a	n/a	n/a	n/a	n/a	n/a
(ref = not a mother)											
**School status**	*In-school*	n/a	n/a	630	7.64 (4.20–13.92)***	575	0.45 (-0.20–1.10)	0.45 (-0.20–1.10)	339	2.20 (1.17–3.22)***	2.20 (1.17–3.22)***
(ref = out of school)											
**Self-efficacy**	*Total score*	630	1.06 (1.02–1.11)**	840	1.04 (1.00–1.08)*	613	0.03 (-0.02–0.07)	0.03 (-0.02–0.07)	381	0.02 (-0.05–0.09)	0.09 (0.23–0.42)
**Caste/ethnicity**	*Brahmin/ Chettri*	255	1.68 (1.13–2.49)**	327	1.41 (0.96–2.06)	244	0.22 (-0.16–0.61)	0.22 (-0.16–0.61)	153	-0.08 (-0.63–0.46)	-0.08 (-0.63–0.46)
(ref = socially-excluded)	*Other*	56	0.66 (0.34–1.29)	71	1.33 (0.68–2.59)	59	-0.50 (-1.21–0.21)	-0.50 (-1.21–0.21)	36	-0.74 (-1.80–0.31)	-0.74 (-1.80–0.31)
**School decision-making input**	*A lot of input*	515	1.44 (0.88–2.34)	n/a	n/a	n/a	n/a	n/a	n/a	n/a	n/a
(ref = none to some input)											
**Going out decision-making input**	*A lot of input*	n/a	n/a	90	0.97 (0.57–1.64)	n/a	n/a	n/a	n/a	n/a	n/a
(ref = none to some input)											
**Health decision-making input**	*A lot of input*	n/a	n/a	n/a	n/a	n/a	n/a	n/a	n/a	n/a	n/a
(ref = none to some input)											
**Food decision-making input**	*A lot of input*	n/a	n/a	n/a	n/a	n/a	n/a	n/a	n/a	n/a	n/a
(ref = none to some input)											
**Household size**	*Total number*	630	0.97 (0.91–1.03)	840	1.00 (0.92–1.09)	613	-0.03 (-0.09–0.02)	-0.10 (0.26–0.70)	381	-0.01 (-0.12–0.10)	-0.03 (0.36–0.30)
**Household head highest completed level of education**	*Primary*	174	1.31 (0.83–2.05)	221	1.46 (0.91–2.34)	168	-0.01 (-0.49–0.47)	-0.01 (-0.49–0.47)	130	0.23 (-0.48–0.93)	0.23 (-0.48–0.93)
	*Secondary or higher*	204	1.27 (0.85–1.91)	264	1.24 (0.82–1.89)	189	-0.04 (-0.49–0.41)	-0.04 (-0.49–0.41)	120	0.34 (-0.33–1.01)	0.34 (-0.33–1.01)
(ref = no (formal) education)											
**Household head sex**	*Female*	292	0.95 (0.64–1.40)	392	0.89 (0.61–1.29)	272	-0.04 (-0.42–0.34)	-0.04 (-0.42–0.34)	166	0.49 (-0.04–1.02)	0.49 (-0.04–1.02)
(ref = male)											
**Household food security**	*Food secure*	387	1.20 (0.81–1.77)	518	1.31 (0.88–1.94)	371	0.24 (-0.15–0.64)	0.24 (-0.15–0.64)	230	-0.04 (-0.62–0.53)	-0.04 (-0.62–0.53)
(ref = food-insecure)											
**Household wealth**	*Total score*	630	1.56 (1.25–1.95)***	840	1.30 (1.07–1.56)**	613	0.41 (0.22–0.60)***	0.48 (0.26–0.70)***	381	0.39 (0.09–0.70)**	0.46 (0.10–0.81)**
**Agro-ecological region**	*Mountains*	105	1.54 (0.88–2.70)	122	1.41 (0.70–2.83)	100	0.21 (-0.43–0.86)	0.21 (-0.43–0.86)	58	0.34 (-0.49–1.18)	0.34 (-0.49–1.18)
(ref = hills)	*Terai*	216	0.55 (0.34–0.91)*	292	0.66 (0.42–1.01)	208	-0.03 (-0.53–0.47)	-0.03 (-0.53–0.47)	137	-0.36 (-1.09–0.37)	-0.36 (-1.09–0.37)
**Disadvantaged community**	*Yes*	202	0.65 (0.42–1.01)	272	0.78 (0.51–1.20)	204	-0.32 (-0.81–0.15)	-0.32 (-0.81–0.15)	112	-0.33 (-0.94–0.28)	-0.33 (-0.94–0.28)
(ref = no)											
(Pseudo) R^2^			0.182		0.30		0.10	0.0		0.13	

Notes: CI or SD in parentheses

***significant p<0.001

**significant p<0.01

*significant p<0.05; n/a indicates not included in analysis

**Table 4:** Regression results for educational, occupational and marital aspirations split by age group.

Aspirations		Education				Occupation				Marriage					
		>class 12 (1) vs. ≤class 12 (0)				Professional skilled job (1)				Age					
						Vs. No/low skilled job (0)									
		N	10–14	N	15–19	N	10–14	N	15–19	N	10–14		N	15–19	
**Observations**		385		245		397		443		358			255		
			aOR		aOR		aOR		aOR		Unstd. B	Std. Beta (95% CI)		Unstd. B	Std. Beta (95% CI)
			(95% CI)		(95% CI)		(95%CI)		(95%CI)		(95% CI)			(95% CI)	
**Age Group**	*15–19 years*	n/a	n/a	n/a	n/a	n/a	n/a		n/a	n/a	n/a	n/a	n/a	n/a	n/a
(ref = 10–14 years old)															
**Maternal status**	*Mother*	n/a	n/a	n/a	n/a	n/a	n/a	443	1.05 (0.47–2.34)	n/a	n/a	n/a	n/a	n/a	n/a
(ref = no children)															
**School status**	*In-school*	n/a	n/a	n/a	n/a	385	8.22 (2.44–27.72)***	245	9.28 (4.57–18.85)***	349	0.02 (-1.47–1.51)	0.02 (-1.47–1.51)	226	0.77 (0.11–1.43)*	0.77 (0.11–1.43)*
(ref = out of school)															
**Self-efficacy**	*Total score*	385	1.10(1.05–1.16)***	245	0.98 (0.92–1.06)	397	1.06 (1.00–1.12)*	443	1.02 (0.96–1.08)	358	0.02 (-0.04–0.07)	0.08 (0.18–0.34)	255	0.02 (-0.05–0.09)	0.09 (0.23–0.42)
**Caste/ethnicity**	*Brahmin/ Chettri*	147	1.63 (0.98–2.70)	108	2.05 (1.07–3.93)*	148	1.24 (0.64–2.41)	179	1.54 (0.94–2.53)	136	0.30 (-0.16–0.76)	0.30 (-0.16–0.76)	108	0.11 (-0.50–0.72)	0.11 (-0.50–0.72)
(ref = socially-excluded)	*Other*	36	0.72 (0.29–1.79)	20	0.81 (0.29–2.27)	39	1.35 (0.57–3.20)	32	1.64 (0.62–4.39)	34	-0.77 (-1.66–0.13)	-0.77 (-1.66–0.13)	25	-0.08 (-0.96–0.80)	-0.08 (-0.96–0.80)
**Input school-related decisions**	*Input in decisions*	303	1.54 (0.86–2.75)	212	1.35 (0.58–3.15)	n/a	n/a	n/a	n/a	n/a	n/a	n/a	n/a	n/a	n/a
(ref = no/limited input)															
**Input in going out & engage in community**	*Input in decisions*	n/a	n/a	n/a	n/a	10	0.76 (0.14–4.18)	80	1.06 (0.57–0.98)	n/a	n/a	n/a	n/a	n/a	n/a
(ref = no/limited input)															
**Input in own health care**	*Input in decisions*	n/a	n/a	n/a	n/a	n/a	n/a	n/a	n/a	n/a	n/a	n/a	n/a	n/a	n/a
(ref = no/limited input)															
**Input in food consumption**	*Input in decisions*	n/a	n/a	n/a	n/a	n/a	n/a	n/a	n/a	n/a	n/a	n/a	n/a	n/a	n/a
(ref = no/limited input)															
**Household size**	*Total number*	385	0.93 (0.87–1.00)*	245	1.05 (0.93–1.18)	397	0.93 (0.87–1.00)*	443	1.07 (0.96–1.19)	358	-0.06 (-0.13–0.01)	-0.17 (0.37–0.03)	255	0.01 (-0.09–0.10)	0.03 (0.25–0.31)
**Household head highest completed level of education**	*Primary*	103	0.85 (0.46–1.56)	71	2.43 (1.10–5.37)*	103	1.89 (0.77–4.61)	118	1.30 (0.71–2.37)	93	-0.22 (-0.37–0.81)	-0.22 (-0.37–0.81)	75	-0.37 (-1.14–0.39)	-0.37 (-1.14–0.39)
	*Secondary or higher*	130	1.17 (0.69–1.98)	74	1.28 (0.64–2.55)	130	1.06 (0.50–2.24)	134	1.45 (0.85–2.47)	115	0.32 (-0.24–0.88)	0.32 (-0.24–0.88)	74	-0.55 (-1.29–0.18)	-0.55 (-1.29–0.18)
(ref = no formal education)															
**Household head sex**	*Female*	191	0.94 (0.56–1.59)	101	0.96 (0.50–1.83)	196	0.80 (0.47–1.36)	196	0.98 (0.59–1.63)	172	0.13 (-0.37–0.63)	0.13 (-0.37–0.63)	100	-0.30 (-1.06–0.46)	-0.30 (-1.06–0.46)
(ref = male)															
**Household food security**	*Food secure*	235	1.42 (0.88–2.31)	152	0.95 (0.47–1.91)	240	0.91 (0.49–1.72)	278	1.65 (0.95–2.88)	216	-0.01 (-0.54–0.56)	-0.01 (-0.54–0.56)	155	0.48 (-0.12–1.08)	0.48 (-0.12–1.08)
(ref = food-insecure)															
**Household wealth**	*Total score*	385	1.40 (1.04–1.88)*	245	1.96 (1.44–2.66)***	397	1.28 (0.97–1.75)	443	1.33 (1.02–1.73)*	358	0.44 (0.19–0.69)***	0.51 (0.22–0.80)***	255	0.37 (0.07–0.67)*	0.43 (0.08–0.78)
**Agro-ecological region**	*Mountains*	53	1.34 (0.70–2.57)	52	2.08 (0.71–6.10)	55	1.78 (0.63–5.07)	67	1.19 (0.51–2.77)	51	0.22 (-0.54–0.98)	0.22 (-0.54–0.98)	49	0.11 (-0.79–1.02)	0.11 (-0.79–1.02)
(ref = hills)	*Terai*	134	0.67 (0.36–1.24)	82	0.36 (0.16–0.80)**	138	0.68 (0.36–1.28)	154	0.59 (0.32–1.08)	120	0.14 (-0.50–0.83)	0.14 (-0.50–0.83)	88	-0.34 (-1.01–0.34)	-0.34 (-1.01–0.34)
**Disadvantaged community** (ref = no)	*Yes*	123	0.69 (0.42–1.14)	97	0.49 (0.21–1.12)	128	0.66 (0.36–1.20)	144	0.90 (0.51–1.59)	119	-0.56 (-1.15–0.04)	-0.56 (-1.15–0.04)	85	0.03 (-0.62–0.68)	0.03 (-0.62–0.68)
(Pseudo) R^2^			0.19		0.18		0.14		0.35		0.12	0.12		0.07	0.07

Notes: CI or SD in parentheses

***significant p<0.001

**significant p<0.01

*significant p<0.05; n/a indicates not included in analysis

### Ethics

Prior written informed consent from all girls and written parental or guardian assent for girls under 16 years of age were obtained. Ethical approval was obtained from the Nepal Health Research Council (No. t97/ZO1B).

## Results

### Sample description

Survey participants were, on average, 15 years old, with slightly more girls in the older age group (52.7%). Almost none of the girls had children (82.1%) and the majority were in-school (75.0%). Self-efficacy scores ranged from 15 to 40, with an average score of 30.7. Almost two-thirds (63.5%) of the girls perceived they had input in most or all education-related decisions and slightly more (69.4%) for decisions related to their own health, whereas only about one in ten (10.7%) reported input into most or all decisions for going out of the house, and less than half (41.8%) for food consumption. Half of the girls belonged to the socially-excluded caste/ethnic groups (52.6%). Total equity scores ranged from -1.64 to 2.68 with a mean of -0.16. Household size ranged from 1 to 37, with a mean total number of household members of 6. About one-third (38.3%) of the households were food insecure. Slightly more than half (53.3%) of the household heads were men and almost half (42.3%) of the household heads had never attended or not completed primary school. Half of the girls resided in the hills (50.7%), 14.5% in the mountains and 34.8% in the plains. One third of the girls were living in areas classified as a disadvantaged community (32.4%) (Table 1).

### Educational aspirations

More than half of the girls (58.6%) aspired to study beyond grade 12. Aspirations for studying beyond class 12 were more prevalent among older adolescent girls (p<0.001), with input into school-related decisions (p<0.001), and higher self-efficacy (p<0.001), as well as those belonging to upper caste (p<0.01) higher economic status (p<0.001), food secure (p<0.05) households with highly educated household heads (p<0.05) and from advantaged communities (p<0.01).

In the fully adjusted logistic regression model, age, self-efficacy, caste/ethnicity, and wealth were positively associated with educational aspirations, whereas residing in the plains was negatively associated. Older girls had 1.7 times greater odds of aspiring to study beyond class 12, compared to younger girls (p<0.01;1.18–2.52). Also, for every unit increase in a girl’s total self-efficacy score, the odds were 1.1 times higher that she aspired to study beyond class 12 (p<0.01; 1.02–1.11). The odds of aspiring to study beyond class 12 were 1.7 times higher among girls belonging to the upper caste/ethnicity groups than those from the socially-excluded groups (p<0.01; 1.13–2.49) and 1.6 times higher with every unit increase (meaning at least 2 additional household assets/structural improvements) in household economic status (p<0.000; 1.25–1.95). Finally, the odds of aspiring higher than class 12 were 0.6 times lower among girls living in the lowland plains (*terai)* compared to those residing in the hills (p<0.05; 0.34–0.91).

For younger adolescent girls’, self-efficacy, household size, and wealth were associated with her educational aspirations. The odds of aspiring to study beyond class 12 were 1.1 times greater for each unit increase in her self-efficacy score (p<0.001; 1.05–1.16) and 1.4 times higher with every unit increase in household equity score (p<0.05; 1.04–1.88). On the other hand, the odds of aspiring to study beyond class 12 were 0.9 times lower with every additional household member (p<0.05; 0.87–1.00). For older girls, household economic status was also associated with educational aspirations, but other determinants included: caste/ethnicity, household head education, and agro-ecological zone of residence. The odds of aspiring to study beyond class 12 were 2.1 times greater among upper caste/ethnic group (p<0.05; 1.07–3.93), and 2.5 times greater if the household head had completed primary education versus no education (p<0.05; 1.10–5.37). Odds increased by 2.0 for every unit increase in total equity score (p<0.001; 1.44–2.66). Older girls living in the *terai* versus in the hills, had 0.4 lower odds of aspiring to study beyond class 12 (p<0.01; 0.16–0.80).

### Occupational aspirations

While three out of four girls aspired to a job that required professional or higher formal education, a quarter aspired to have no job or a job requiring lower or no (formal) education. Aspirations for occupations requiring higher education were more prevalent among girls who were older (p<0.001), without children (p<0.001), school-going (p<0.001), with input into decisions about their mobility (p<0.05), and higher self-efficacy scores (p<0.001), as well as among those belonging to households from upper caste/ethnic groups (p<0.05) and with higher economic status (p<0.05).

In the fully adjusted logistic regression model, only school status, self-efficacy, and household economic status were significantly associated with occupational aspirations. School-going girls had 7.6 times higher odds than out-of-school girls to aspire to an occupation requiring higher education (p<0.000; 4.2–13.9). The odds of aspiring for occupations requiring higher education were only 1.04 times greater with every unit increase in girls’ self-efficacy score (p<0.05; 1.0–1.08) and 1.3 times higher with every unit increase in total equity score (p<0.01; 1.07–1.56).

School status was highly associated with aspiring for occupations with higher educational requirements in models for both younger and older adolescents. The odds of having such aspirations were 8.2 (p<0.001; 2.44–27.72) and 9.3 (p<0.001; 4.58–18.85) times higher among in-school versus out-of-school girls, among younger and older girls respectively. For younger adolescent girls, self-efficacy and household size were also associated with occupational aspirations. With every unit increase in a girl’s total self-efficacy score, she had 1.1 times greater odds of aspiring for a job with higher educational requirements (p<0.05; 1.00–1.12), and with every additional household member, these odds were 0.9 lower (p<0.05; 0.87–1.00). For older adolescent girls, in addition to going to school, household economic status had a positive association with occupational aspirations requiring higher education. With every unit increase in wealth score, girls had 1.3 times greater odds of aspirations for occupations with higher educational requirements (p<0.05; 1.02–1.73).

### Marital aspirations

The aspired age of first marriage for unmarried girls ranged from 15 to 32 years, with a mean aspired age of marriage of 22 years. Girls who were older (p<0.01), with higher total self-efficacy scores (p<0.01), and from households with better economic (p<0.001), and food security status (p<0.01), in the lowland plains or hills compared to the mountains (p<0.05), and from disadvantaged communities (p<0.001) aspired to marry at later ages (p<0.01).

In the fully adjusted multiple linear regression models, only age group and household economic status were significantly associated with marital aspirations. Being an older adolescent was associated with aspiring to marry at half a year later (B = 0.52, p<0.01; 0.16–0.88) and each unit increase in the household’s equity score was also associated with nearly a half year increase in aspired age of marriage (B = 0.41, p<0.000; 0.22–0.60).

For younger girls, only household economic status was significant, which was associated with nearly a half year increase in aspired age of marriage (B = 0.44, p<0.001; 0.19–0.69). Among older girls, household economic status was associated with a one third year increase in aspired age of marriage (B = 0.37, p<0.05; 0.07–0.67), and going to school was associated with a half a year increase in aspired age of marriage (B = 0.41, p<0.000; 0.22–0.60).

### Fertility aspirations

Less than half of the girls (43.3%) had undecided fertility aspirations and 1.5% did not wish to have any children. Among those with fertility aspirations, the preferred age of having a first child ranged from 18 to 35 years of age, with a mean age of 24. Later aspired ages of having a first child were more prevalent among school going girls (p<0.001), as well as among girls from households with greater economic status (p<0.01), female-headed households (p<0.05), and living in more advantaged communities (p<0.05).

In the fully adjusted models, only school status and household economic status were associated with girls’ aspired age of having a first child. Going to school was associated with aspiring to have a first child more than 2 years later compared to not attending school (B = 2.2, p<0.000; 1.17–3.22). Every unit increase in household wealth was associated with a more than one third increase in aspired age of having a first child (B = 0.39, p<0.01; 0.09–0.70).

### Health and nutrition aspirations

Almost all adolescent girls indicated they perceived health and nutrition as very important: 90.2% and 89.8% respectively. Higher health aspirations were more prevalent among girls who had input in health-related decisions-making (p<0.05), who came from households that were economically better off (p<0.001), as well as food secure (p<0.05). Higher nutrition aspirations were also more prevalent among girls that had input in food-related decision-making (p<0.001), higher self-efficacy scores (p<0.01), and from households with better economic status (p<0.05) and residing in advantaged communities (p<0.05).

## Discussion

This study assessed adolescent girls’ (10–19 years) aspirations in several life domains—education, occupation, marriage, fertility, health, and nutrition- and estimated associations between various individual, household, and community characteristics with each of these aspirations, overall, and separately for younger (10–14 years) and older (15–19 years) girls in Nepal. More than half of the girls aim to continue their studies beyond grade 12; three-quarters of the girls aspire a job requiring professional or higher formal education; aspired ages of marriage and fertility were above the national and global recommendations of 20 years; and almost all girls perceived health and nutrition as very important in their lives. We found variation in which individual, household, and community factors were associated with aspirations by domain, and that determinants of aspirations in a specific domain differed between younger and older girls.

Findings confirm that girls’ capacity to aspire is not homogeneously distributed [25, 31]. Household wealth was strongly associated with girls’ aspirations in domains of education, occupation, fertility, and marriage. This finding implies that girls living in less wealthier households have lower aspirations. Although a few studies conducted in LMICs, including Nepal, found that girls kept relatively high aspirations especially related to education, but also careers and family life throughout adolescence and despite poverty [4, 47, 49], our findings are consistent with evidence on hopelessness, fatalism and lower aspirations among poorer populations [25], and literature on the psychology of aspirations and the culture of poverty [25, 29, 71]. A girl living in a poorer household, is more likely to internalize feelings of hopelessness and has lower aspirations. It is also highly likely that these girls entered adolescence already having accumulated (intergenerational) disadvantage [47]. When poverty distracts from setting goals for the future, this leads to a so-called poverty trap. Moreover, living up to socio-cultural norms and seeing peers or parents making decisions that do not change their lives has been shown to lead youth to set the same aspirations [72]. Age-specific findings imply however that wealth did not (yet) influence younger girls’ occupational aspirations. Their aspirations may be unrealistically high [13]. It is likely that, at this stage, aspirations are shaped without paying too much attention to the ability and reality to realize aspirations, and are largely influenced by for others in their aspiration window, such as peers [3, 24]. The potential intergenerational cycle of low aspirations and poverty reinforcing each other, especially visible for older girls, is an unfortunate dynamic [24]. Findings suggest that to foster, particularly older girls’ aspirations, economic opportunities, social protection, high-quality education, and other initiatives that address underlying poverty and other context-specific factors are needed.

We found no significant associations between girls’ aspirations and the head of households’ sex, household food security, or living in a disadvantaged area. While these findings may suggest a limited influence of household and the community factors, girls from the upper caste/ethnic group, however, had higher odds of aspiring to study beyond class 12. This association mattered more for older girls, and relates to the fact that while secondary school enrolment is now the norm in Nepal, the most disadvantaged girls still face barriers to education [73]. This finding stresses the importance for programs to prioritizing girls from socially-excluded groups vis-a-vis education investments, as well as understanding and addressing socio-cultural barriers that hinder girls from lower caste/ethnic groups to aspire to higher education. Moreover, where the head of a household completed at least primary education, girls’ educational aspirations were higher. This association could be related to higher parental encouragement and support, which is in line with findings from previous studies [74]. The finding may also imply that engaging household decision-makers, and parents, is important for fostering adolescent girls’ aspirations. We also found that household size was negatively associated with younger girls’, but not older girls’ educational and occupational aspirations. This could be explained by the resource dilution theory that poses that parental or household resources and time are finite [75]. The theory may partly explain lower aspirations of younger girls, as the more siblings one has, the fewer resources are available to them. For instance, a multi-country study using survey data from over 168,000 children showed that in India, intrafamily decision-making resulted in unequal allocations and accompanying disadvantages in child health. The study provided evidence for height and health care inequalities across birth order, which were more severe in families with higher son preference [76]. This could potentially result in younger girls’ lower aspirations. Future research on the role of sibling configurations and family nature, including the number of sons and daughters, their sex and birth order in shaping adolescent aspirations, has yet to shed more light on this.

Findings particularly highlight that aspiration formation is dynamic [3, 47]. Whilst adolescence is commonly referred to as a distinct period from child- or adulthood, results show that aspirations vary by stages of adolescence. In contrast to other studies that show a (negative) adjustment of aspirations over time [31, 77], some aspirations seemed to increase with age in Nepal. This is in line with studies showing that girls keep relatively high aspirations, related to education, careers and family life, despite perceiving this as a challenging time [47]. Moreover, the high percentage of girls that perceived health and nutrition as important, combined with the high mean of aspired ages of marriage and having a first child are hopeful and may indicate social change, or changing norms and changing practices. Older girls aspired for higher educational levels and later ages of marriage than younger girls. This may not only reflect changing norms surrounding girls’ expected life trajectories but could also indicate that girls’ achievements so far enabled them to aspire higher.

The high prevalence of uncertain fertility and marital aspirations might reflect the fact that while childhood and early adolescence are characterized by being present-oriented with little interest in the future, during adolescence goal-setting capacity increases [13, 78]. Younger girls discuss different goals, needs, and aspirations than older adolescent girls. It is thus unsurprising that the younger girls did not think about marriage and fertility as much as the older girls. Younger girls’ potentially unrealistically high occupational aspirations, on which household economic status did not seem to have an effect, may also relate to the inability to think about their life that far down the road. Considering these findings, uncertain or undecided aspirations could be highly interesting as they may influence future outcome. Yet, they are rarely studied in LMICs. The scarce evidence coming from high-income contexts showed for instance that uncertain educational aspirations were associated with lower educational attainment [79]. It might be well the case that revealing and talking about aspirations is a sensitive topic and might be considered taboo in the context of Nepal. As from this particular study it remains unclear why some girls do not know what their aspirations are regarding family formation, understanding uncertain or undecided aspirations in low-resource contexts, besides the effect of age, would be an area of future, preferably mixed-methods, research.

Findings strongly underscore the role of schools in shaping aspirations. Staying in school and thereby delaying marriage might lead to higher educational, as well as marital and fertility aspirations. School participation broadens girls’ horizons and is a major determinant of aspirations in several life domains, which seems to become even more important as girls grow older [62]. Schools have the potential to play a role in aspiration formation and goal-setting by, for instance, providing realistic and tailored information. Although girls’ secondary school enrolment has never been higher in Nepal, the next steps are to ensure quality education, to help girls pursue further education, and to create educational and training opportunities for out-of-school- and married girls as well. These findings also imply interrelations between aspiration domains (e.g., between educational and occupational aspirations, or marital and fertility aspirations), and although beyond the scope of the present study, future research should further examine these interrelations.

Despite being known as building blocks of aspirations, self-efficacy, and girls’ perceived input in decision-making in key life domains, both reflecting the extent to which a girl feels able to influence her own life, were only modestly associated with, only educational and occupational aspirations. Our findings, are in line with previous studies showing self-efficacy as one of the key determinants of these aspirations in particular [41, 80]. However, findings showed that self-efficacy matters mostly for younger girls. It is therefore vital to invest in age-tailored programs and policies that raise socio-emotional skills from early ages onwards. Low self-efficacy in this age group, might lead to an aspirations gap, which implies that when the gap between a girl’s current living situation and her aspiration becomes too big, it will lead to a feeling of unreachable goals, frustration, or hopelessness [24].

### Strengths, limitations, and future research

Our study advances previous research in multiple ways. First, this is, to our knowledge, the first empirical study in South-Asia, or in any low-income context, that examines the nature and determinants of a range of girls’ aspirations in several important domains. We, however, relied on separate measures to estimate aspirations, and used a non-standardised proxy for health and nutrition aspirations (perceived importance). While we assumed high perceived importance to lead to higher aspirations, the high rates of high perceived importance (90%) left us unable to estimate regression models. In this respect, further research should invest in exploring and validating ways to measure adolescents’ aspirations in health and nutrition. Second, our study used a large dataset that is close to being nationally representative. Additionally, the dataset included the entire adolescent range of 10 to 19 years, whereas most studies focus on a specific age- or life stage; the disaggregated analyses provide a more nuanced picture of aspirations, and how determinants of aspirations differ between younger and older girls. However, for our age-group-specific models, the sample sizes become relatively small, and there may not be power to detect significance for all factors. Since we did not interview out-of-school girls, married girls, or mothers about their (past) aspirations related to school, marriage, or fertility respectively, due to ethical concerns, our sample sizes for each of the aspiration domains differed and results should thus be interpreted with caution. Resulting avenues for future research could be to explore out-of-school girls’ educational aspirations, or their “past” aspirations and its determinants. Moreover, while we acknowledge that the transition into adulthood is not linear and aspirations are likely shaped over time, the cross-sectional design prevents us from assessing this time variation or establishing causality between (changes in) factors such as household wealth and aspirations. Therefore, future reporting from this longitudinal study and other similar research will better enable a life course perspective. Finally, despite including proxies of social influence (i.e. household head’s highest completed level of education, household head sex), and individual-level decision-making power which could be a result of gendered norms, aspirations are shaped in interaction with others and are influenced by prevailing socio-cultural norms in families, households, and communities [5, 24]. Therefore, we recommend future research to include other measures of prevailing socio-cultural norms at the community level, such as women’s autonomy indicators, as well as to consider the importance of the nature and composition of the family, such as extended versus nuclear families, birth order, and sibling configuration. Finally, future research should include indicators of social processes and influences such as parental expectations or the roles of important others in shaping girls’ aspirations.

### Conclusion

Adolescence is a period wherein concerns about one’s future are greater than in any other developmental phase [81], and when aspirations can be powerful drivers of adolescent wellbeing and human development. Findings underscore the need to foster and invest in the development of girls’ aspirations throughout different stages of adolescence and consider the multileveled and context-specific and structural factors. Herein, heterogeneity among adolescent girls should be acknowledged and further researched, including differences between younger and older girls, school-going girls and out-of-school girls, married and unmarried girls, adolescent mothers and those without children, and those from advantaged and disadvantaged socio-economic backgrounds in order for aspirations to become road maps to help girls thrive.
